# The relationship between organ-tissue body composition and resting energy expenditure in prepubertal children

**DOI:** 10.1038/s41430-018-0344-2

**Published:** 2018-10-22

**Authors:** Taishi Midorikawa, Yuki Hikihara, Megumi Ohta, Takafumi Ando, Suguru Torii, Shizuo Sakamoto, Shigeho Tanaka

**Affiliations:** 1 College of Health and Welfare, J.F. Oberlin University, 3758 Tokiwamachi, Machida Tokyo, 194-0294 Japan; 2 Waseda Institute for Sport Sciences, Waseda University, 2-579-15 Mikajima, Tokorozawa Saitama, 359-1192 Japan; 3 Department of Nutrition and Metabolism, National Institute of Health and Nutrition, National Institutes of Biomedical Innovation, Health and Nutrition, 1-23-1 Toyama, Shinjuku-ku Tokyo, 162-8636 Japan; 4 Faculty of Creative Engineering, Chiba Institute of Technology, 2-1-1 Shibazono, Narashino Chiba, 275-0023 Japan; 5 School of International Liberal Studies, Chukyo University, 101 Tokodachi, Kaizu-cho, Toyota, Aichi 470-0393 Japan; 6 Faculty of Sport Sciences, Waseda University, 2-579-15 Mikajima, Tokorozawa Saitama, 359-1192 Japan

**Keywords:** Nutrition, Weight management, Paediatrics

## Abstract

**Background/Objectives:**

In this study, we ascertained the relationship between resting energy expenditure (REE) obtained using two procedures: indirect calorimetry and from organ-tissue mass, calculated employing magnetic resonance imaging (MRI), and adult metabolic rate constants, in prepubertal children. Differences between the measured and the calculated REEs were assessed according to age at puberty approaching stage.

**Subjects/Methods:**

We recruited 6–12 years old 110 healthy Japanese prepubertal children (40 girls and 70 boys). Organ-tissue masses for different organs (skeletal muscle, liver, kidneys, brain and adipose tissue) were determined using MRI and dual-energy X-ray absorptiometry. Heart and residual masses were calculated on the basis of each equation. REE was measured using the Douglas bag technique (measured REE). On the other hand, calculated REE was obtained by multiplying the sum of body compartments with the corresponding adult tissue respiration rate.

**Results:**

The measured REE was significantly greater than the calculated REE in both, boys and girls, although a significant association was noticed between the two REEs in both the sexes. Besides, correlation between age and difference in the two REEs was found to be significant only in girls.

**Conclusions:**

The present study revealed that: (1) measured and calculated REEs differ by approximately 300 kcal/day in a relatively large sample of prepubertal children, and (2) the difference in organ-tissue mass between the measured and calculated REEs increased from approximately 200 to 400 kcal/day during the developmental process in girls but not in boys.

## Introduction

Children have lower body mass and fat-free mass (FFM) than adults, but their absolute resting energy expenditure (REE, kcal/day) is comparable with that in adults. However, the proportion of REE to body mass decreases from approximately 30–40 kcal/(kg day) at 6–12 years to 26 kcal/(kg day) at 18 years [1]. An in-depth knowledge regarding the association between body composition and REE might yield valuable information regarding energy requirements and weight management in children.

The variation in REE is due to variable organ-tissue mass [2–4]. The organ-specific resting metabolic rate (e.g., 13 kcal/[kg day] for skeletal muscle (SM), 200 kcal/[kg day] for the liver, and 440 kcal/[kg day] for the kidney) remains almost constant in young, as well as middle-aged women and men, and underweight and obese healthy adults [2–4]. Moreover, a previous study reported that the high REE (i.e., mean measured REE, 2286 kcal/day) seen in sumo wrestlers is probably not because of an elevated organ-tissue metabolic rate but due to an increased volume of tissue with low- and high-metabolic activity [5]. According to a recent study, training for aerobic endurance has no role to play in the chronic increase in adult resting metabolic rate, in people with a VO_2_peak of about 60 ml/(min kg) [6]. Although the individual variation is minimal, the organ-tissue metabolic rate would be almost constant for the variability in adults.

By contrast, the organ-tissue metabolic rate for children was not indicated in previous studies. The only previous study performed in children indicated that REE, using adult resting metabolic rate, was underestimated when compared with the REE measured using indirect calorimetry (i.e., measured REE, 1224 kcal/day vs. calculated REE, 925 kcal/day) [7]. Hence, the organ-specific resting metabolic rate in children is likely higher than that in adults. However, a drawback of the previous work was the small number of subjects (eight boys and seven girls). Therefore, the present study focused to reaffirm the relationship between the measured and calculated REEs in a relatively bigger group of prepubertal children, using a previously published approach [2, 7]. In addition, whether the changes in the differences in measured and calculated REEs according to age would be observed at the puberty approaching stage, is unknown. To attain the second study objective, we assessed this relationship in prepubertal children.

## Materials and methods

### Subjects

We recruited 110 healthy Japanese prepubertal children (70 boys and 40 girls) in the 6–12 years age group, who were at Tanner stage 1 in this study, via recommendations from friends and contacts in Tokyo. The research staff assessed the maturation level of all the participants aged 10–12 years (47 boys and 22 girls), employing the Tanner scale questionnaire [8]. The present study’s subjects did not comprise of any players, who performed a specialist exercise training. There were no known pathologies or use of medications by any of the recruited boys or girls. There were no additional criteria for inclusion of subjects (such as demographic and socioeconomic status). All the methods involving human subjects were approved by the ethical committees of National Institute of Health and Nutrition and Waseda University, and the guidelines of the Declaration of Helsinki were followed in this study. All the participants and their guardians provided written informed consent. All questionnaire, anthropometric, body composition, and REE data were collected during a single-study visit in Waseda University. REE was the first-order measurement, and the anthropometric and body composition measurements were appropriately assigned after measuring REE and having a small snack (i.e., energy jelly).

### Measurements by anthropometry and dual-energy X-ray absorptiometry

Body mass was determined to the accuracy of 0.1 kg by using the same calibrated digital balance (DC-320, TANITA Co. Ltd), and measurement of height was done to the accuracy of 0.1 cm with the same calibrated portable stadiometer (YS-OA, AS ONE Co. Ltd) for all the subjects, dressed in minimal clothing, without footwear. Body mass index was determined by the formula, body weight (in kg)/square of the height (in meters) (kg/m^2^; Table 1). FFM and total fat mass were assessed with dual-energy X-ray absorptiometry (DXA; Delphi A-QDR, Version 12.4.3 Pediatric Whole body, Hologic Inc., Bedford, MA, USA; Table 1). The estimated coefficient of variation (CV) was < 1% based on the intraobserver variability for assessing fat mass and FFM, employing DXA from the test–retest analyses done previously. The anthropometric measurements, using DXA and magnetic resonance imaging (MRI), were made on the same day.

**Table 1:** Subject characteristics

	Boys			Girls		
	*n* = 70			*n* = 40		
Age (year)	10.0	±	1.7	9.7	±	1.7
Standing height (cm)	137.3	±	11.5	136.0	±	11.7
*Z*-score	0.3	±	1.0	0.2	±	1.2
Body mass (kg)	33.8	±	10.0	32.7	±	9.2
*Z*-score	0.4	±	1.4	0.4	±	1.6
BMI (kg/m^2^)	17.5	±	2.9	17.4	±	2.9
Fat (%)	24.1	±	7.6	28.2	±	7.1
Fat mass (kg)	8.6	±	5.0	9.5	±	4.3
Fat-free mass (kg)	25.2	±	6.0	23.2	±	6.0

Body fat percentage, fat mass and fat-free mass were measured using dual-energy X-ray absorptiometry

Standing height and body mass are calculated as *Z-*scores using the physical fitness standards for Japanese people [18]

*BMI* body mass index

### Measurement of organ-tissue mass using MRI

The whole-body quantities of liver, kidney, the brain and SM were estimated using a General Electric Signa EXCITE VI 1.5 Tesla scanner (Milwaukee, WI, USA). Details of the MRI procedures were as detailed before [6, 9]. Briefly, a T1-weighted spin-echo, axial-plane sequence was used, with a 500-ms repetition time and 13.1-ms echo time, during breath-holding and normal breathing scans. We obtained contiguous transverse images with a 1.0-cm slice thickness (0-cm interslice gap) from the top of the head to the malleolus lateralis of each subject. A highly trained technician traced all the images (approximately 100–150 slices per person) with hand-derived calculations. The MR images analyzing included brain, SM, and abdominal organ segments, but excluded blood vessels, connective tissue, and fat tissue. The ZedView software (LEXI Co., Ltd, Tokyo, Japan) was used to analyze the MRIs for segmentation and assessment of the tissue cross-sectional areas.

The volumes of liver, kidney, brain, and the SM were determined from the sum of the cross-sectional area (cm^2^), measured by the MRI tracing, followed by the multiplication of the area with the slice thickness (1 cm). Employing the tissue densities given below, the recorded tissue volumes (cm^3^) were converted to masses (kg): SM: 1.041 g/cm^3^ [10]; liver: 1.060 g/cm^3^; kidney: 1.050 g/cm^3^; and the brain: 1.036 g/cm^3^ [11]. The estimated CV was 2%, for the measurements of SM volume, from a test–retest analysis [9]. The percentages of the differences in measurements, done by the same technician, on two separate days for the same scan, were 0.3, 0.5, and 0.6%, for liver, kidney, and the brain, respectively (*n* = 5).

As the continuously beating heart led to artifacts, the heart mass (g) was deduced from the height and body mass, with the formula: 22.81 × height (m) × body mass^0.5^ (kg) − 4.15 for boys and 19.99 × height (m) × body mass^0.5^ (kg) + 1.53 for girls [12], which was based on the regression line from Japanese autopsy data for ages 0–95 years (1914 males and 1223 females). Adipose tissue mass was determined from fat mass measured on DXA, with the belief that 85% of the adipose tissue was fat, whereas the remaining 15% was consisted as the calculated component of fat-free adipose tissue (i.e., the difference between adipose tissue mass measured by MRI, and fat mass measured with DXA) [13]. DXA was the first-choice modality for estimating adipose tissue mass in the view of a short analysis time. Residual mass was estimated by subtracting the sum of the masses of the SM, adipose tissue, brain, liver, kidney, and heart from the total body mass, because of the assumption that the total body mass includes all the masses of the organs. Thus, the residual mass consists of skin, bone, connective tissue, intestine, blood, and lung tissue [10].

### Measurement of REE

Open-circuit indirect calorimetry was used to measure REE with the Douglas bag technique [14]. Twelve hours prior to the measurements, there was no consumption of any liquids, except water or food by the subjects. Also, 36 h prior to the measurements, neither of the participants conducted any physical exercise. The participants were advised to reduce physical exertion prior to the assessment of REE and they used a vehicle for traveling from their home to the laboratory. We performed all REE determinations done in the morning were between 07:30 and 10:00 h. Once they arrived in the laboratory, the participants rested in the supine position for half an hour, and had a face mask (Vise Medical, Japan) attached (i.e., at 0–25 min: the subject rested supine on a mat, at 25–30 min: the face mask was attached, at 30–40 min: gas collection time 1, and at 40–50 min: gas collection time 2). We collected expired air for 10 min × 2 times and used the average value for further analysis. While conducting the measurements, the room temperature was kept stabilized (20–25 °C), with minimal noise. We instructed the participants to stay alert, quiet, and without moving prior to and during the measurements. An O_2_ and CO_2_ analyzer (AE-300S, Minato Medical Science, Japan) was employed to analyze the rates of O_2_ consumption and production of CO_2_. A dry gas volume meter (DC-5, Shinagawa, Japan) was used to determine the volume of expired air, which was expressed for conditions of standard temperature, pressure, and dry gas. Weir equation was used to transform the gas exchange results to REE (kcal/day) [15]. The mean CVs (*n* = 5) based on the test–retest assessments, were 3.6%, 3.8%, 3.7%, and 5.2%, respectively, for REE, VO_2_, VCO_2_, and respiratory quotient.

### Calculation of REE

REE was calculated as the sum of seven body compartments (adipose tissue, SM, heart, brain, kidney, liver, and residual mass) multiplied by the respiration rate of the respective tissue, which was based on the reported specific tissue metabolic rates [16]. To compute the calculated REE, we employed the equation [2] : calculated REE (kcal/day) = (4.5 × adipose tissue mass) + (13 × SM mass) + (200 × liver mass) + (240 × brain mass) + (440 × kidney mass) + (440 × heart mass) + (12 × residual mass).

### Statistical analyses

The results are presented as mean ± standard deviation (SD) for all variables. The difference between the measured and calculated REEs was examined using a paired *t*-test. The Spearman rank correlation coefficient and Pearson product-moment correlation analysis were conducted to compare the calculated and measured REE after performing a normality test (i.e., Kolmogorov–Smirnov test). Bland–Altman analysis was used by graphing the differences in the calculated and measured REE values, against the respective averaged values [17]. The relationship of age with the differences in measured and calculated REEs for all boys and girls was assessed using the Spearman rank correlation coefficient. Statistical analyses were used by SPSS version 23.0 for Windows (IBM SPSS; SPSS Inc., Chicago, IL, USA). Differences were considered to be statistically significant at *p* < 0.05.

## Results

**Fig. 1:**
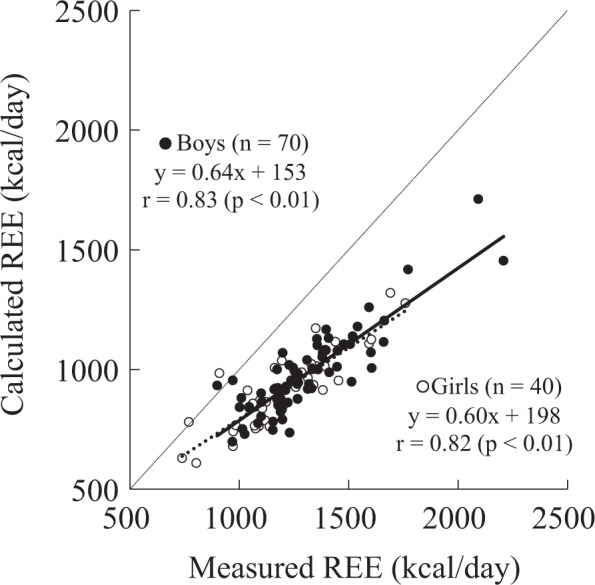
Relationship between measured and calculated resting energy expenditures (REEs). Solid circle and solid line: boys (*n* = 70), open circle and dot line: girls (*n* = 40)

**Fig. 2:**
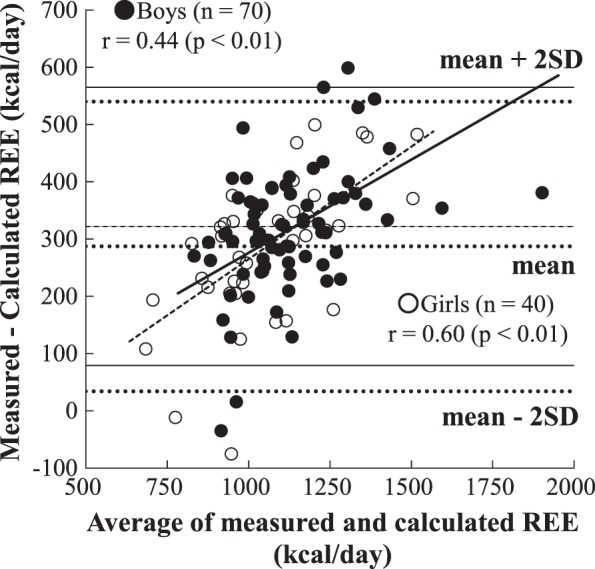
Bland–Altman analysis for comparing measured and calculated resting energy expenditures (REEs). Solid circle and solid line: boys (*n* = 70), open circle and dot line: girls (*n* = 40)

**Fig. 3:**
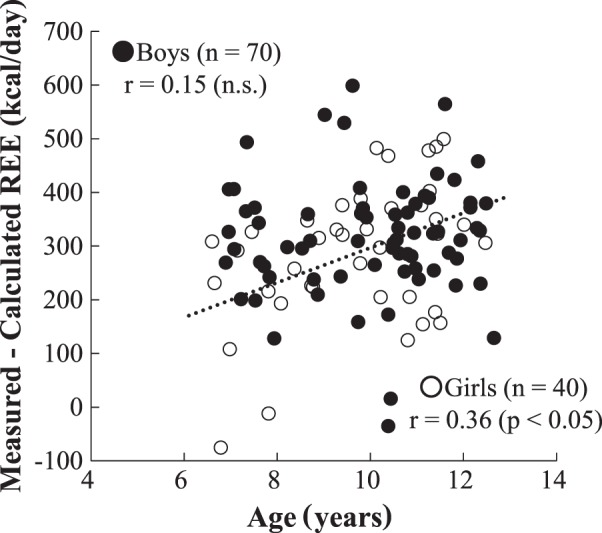
Relationship between age and the differences in measured and calculated resting energy expenditures (REEs). Solid circle: boys (*n* = 70), open circle and dot line: girls (*n* = 40)

The physical characteristics of the participants and their organ-tissue masses are summarized in Tables 1 and 2. The mean standing height and weight were comparable with the standards of physical fitness for Japanese people [18]. The subjects included 11 overweight boys, 2 obese boys, and 12 overweight girls according to BMI categories [19]. The girls had approximately 4% higher body fat percentage and 1.3 kg lower SM mass than the boys. The measured REE (1302 kcal/day for boys and 1200 kcal/day for girls) was significantly (*p* < 0.01) higher than the calculated REE (980 kcal/day for boys and 913 kcal/day for girls) in both the sexes, and the difference between the measured and calculated REE was approximately 300 kcal/day (Table 3). A significant association between the measured and calculated REEs was noticed in the two groups (*r* = 0.83, *p* < 0.01 for boys and *r* = 0.82, *p* < 0.01 for girls) (Fig. 1). A significant trend was observed using a Bland–Altman plot in both the boys (*r* = 0.44, *p* < 0.01) and girls (*r* = 0.60, *p* < 0.01; i.e., a higher overestimate from the higher mean REE; Fig. 2). The Bland–Altman plot indicated a bias of 322 kcal/day with 95% limits of agreement of 79–565 kcal/day for boys and 287 kcal/day with 95% limits of agreement of 34–540 kcal/day for girls (Fig. 2). Additionally, age was significantly correlated with the differences in measured and calculated REEs in the girls but not in the boys (Fig. 3).

**Table 2:** Organ-tissue body composition

Organ-tissue mass (kg)	Boys			Girls		
	*n* = 70			*n* = 40		
Skeletal muscle	9.5	±	2.8	8.2	±	2.5
Adipose tissue^a^	12.4	±	5.6	13.0	±	4.8
Liver	0.92	±	0.28	0.87	±	0.23
Brain	1.48	±	0.12	1.34	±	0.12
Heart^b^	0.18	±	0.04	0.16	±	0.03
Kidney	0.17	±	0.04	0.17	±	0.05
Residual^c^	9.2	±	2.2	9.0	±	2.6

^a^Assumed that 85% of adipose tissue is fat and 15% of adipose tissue is the remaining calculated fat-free component [13]

^b^Boys (22.81 × height (m) × body mass^0.5^ (kg) − 4.15)/1000; Girls (19.99 × height (m) × body mass^0.5^ (kg) + 1.53)/1000 [12]

^c^Residual mass was calculated as body mass minus sum of other measured mass components

**Table 3:** Measured and calculated resting energy expenditure

	Boys			Girls		
	*n* = 70			*n* = 40		
Measured REE (kcal/day)	1302	±	233	1200	±	237
Calculated REE (kcal/day)	980	±	172	913	±	163
Difference (measured–calculated)	322	±	121*	287	±	126*

*REE* resting energy expenditure

*p < 0.01: measured REE compared with calculated REE

## Discussion

In the present study, we observed a difference of approximately 300 kcal/day between the measured and calculated REEs, which were unadjusted and mean absolute values, in prepubertal children. The organ-tissue metabolic rate in prepubertal children has been indicated to be higher than that in adults. These findings, including a numerical difference value in the present study (i.e., a difference of 309 kcal/day between the mean measured REE, 1265 kcal/day and the calculated REE, 956 kcal/day, for 70 boys and 40 girls aged 9.9 years), matched the results obtained in the only previous study (i.e., a difference of 299 kcal/day between the mean measured REE, 1223 kcal/day and the calculated REE, 925 kcal/day, for 8 boys and 7 girls aged 9.3 years) [7]. Therefore, these results indicated the following formula: measured REE in prepubertal children (kcal/day) = calculated REE in prepubertal children (kcal/day) using organ-tissue masses derived from MRI and adult organ-tissue metabolic rates (kcal/[kg day]) + approximately 300 kcal/day, which would be a growth-related energy. This information regarding the energy requirements and weight management in children should be useful.

Another observation of this study was that the difference between the measured and calculated REEs according to organ-tissue masses increased from approximately 200 to 400 kcal/day during prepubertal growth, that is, from ages 6 to 12 years, in girls but not in boys. The results showed that the changes in the differences in measured and calculated REEs according to age would be observed at the puberty approaching stage. In general, height velocity peaks earlier in girls than in boys for 2–3 years [20]. Although the underlying reasons for this phenomenon in children have not been clarified yet, one potential explanation may be related to the age at which puberty begins, and the bone and muscle cells have been speculated to proliferate rapidly in girls. Moreover, this phenomenon is also expected to occur in boys by the time they reach 15 years old.

As far as we know, the organ-tissue metabolic rate for children was not indicated in previous studies. Moreover, we were unable to estimate which organ-tissue has higher metabolic rate in children than in adults by using the approach in the present study. By using positron emission tomography, the previous study pointed out that during growth, the brain’s metabolic rate is not constant [7]. In addition, it is speculated that the metabolic rates in bones, muscles, and internal organs temporarily elevate in children because the number of cells for these organ-tissue dynamically increases during puberty maturation. Further studies are required to ascertain whether each organ-tissue metabolic rate changes in children by using noninvasive methods.

Information regarding SM (i.e., low metabolic organ-tissue) mass in children is limited. Previous MRI studies reported an average mass of 12.2 kg [7] and revealed that the whole-body SM mass in prepubertal children was half that in adults [9, 21, 22]. By contrast, information regarding internal organ (i.e., high-metabolic organ-tissue) mass in prepubertal children in vivo is extremely rare. The present work indicates that the liver mass (0.92 kg for boys at an average age of 10.0 years and 0.87 kg for girls at a mean age of 9.7 years) measured using MRI was similar to the human autopsy data for Japanese people (0.95 kg for boys aged 10 years and 0.86 kg for girls aged 9 years) and was three-fifths that in young adult men (1.40 kg) [5, 12]. The kidney mass (0.17 kg for both boys and for girls) was similar to the human autopsy data (0.17 kg for boys and 0.16 kg for girls), and was half that in adults (0.33 kg) [5, 12]. The results of the only previous study (liver mass of 0.89 kg and kidney mass of 0.18 kg) [7], the human anatomy data, and the present study suggest that internal organ mass gradually increases at the puberty approaching stage.

The measurement of body composition has possible limitations. We recognized that to precisely measure the masses of the brain, liver, and kidneys, contiguous transverse images with < 0.5-cm slice thicknesses are needed. In fact, previous MRI studies in children used the sequence with a 0.5-cm slice thickness and no interslice gap for measuring internal organs, although the slice thickness was at 1.0 cm intervals for 3.5 cm for estimating whole-body SM [7]. MRI with a 1.0-cm slice thickness and 0-cm interslice gap in the present study is also enough to accurately estimate organ-tissue volume, which should provide the excellent distribution curve of whole-body SM volume, as described by previous study [9]. Ideally, the heart mass should be more correctly estimated by such as using electrocardiogram-triggered MRI (with an assumed metabolic rate of 440 kcal/[kg day]). However, we have decided that the reduction of overload and burden along with experiments should also be the first preferred choice for children. Second, children had a small snack for preventing low glucose levels before DXA and MRI measurements, which might have affected the organ-tissue volume, especially that of the liver.

In conclusion, the present study revealed that (1) a difference of approximately 300 kcal/day was detected between the measured and calculated REEs in a relatively large sample of prepubertal children, and (2) the difference between the measured and calculated REEs according to organ-tissue masses increased from approximately 200 to 400 kcal/day during the developmental process in girls but not in boys. On the basis of the present study findings of higher organ-tissue metabolic rate and smaller organ-tissue mass in prepubertal children than in adults, we could clarify why REE in children is comparable with that in adults. This valuable information might well contribute to the energy requirements and weight management in children.
